# Supplementary material for: Causal Relationship between Aging and Anorexia Nervosa: A White-Matter-Microstructure-Mediated Mendelian Randomization Analysis
Source: Biomedicines. 2024 Aug 16;12(8):1874. doi: 10.3390/biomedicines12081874 (PMC11351342; doi:10.3390/biomedicines12081874)
Supplement: Supplementary file 1 [file biomedicines-12-01874-s001.zip › Supplementary Figure/Figure S4/Singe SNP plot of MR analysis for TL to ubm-b-1848.pdf]

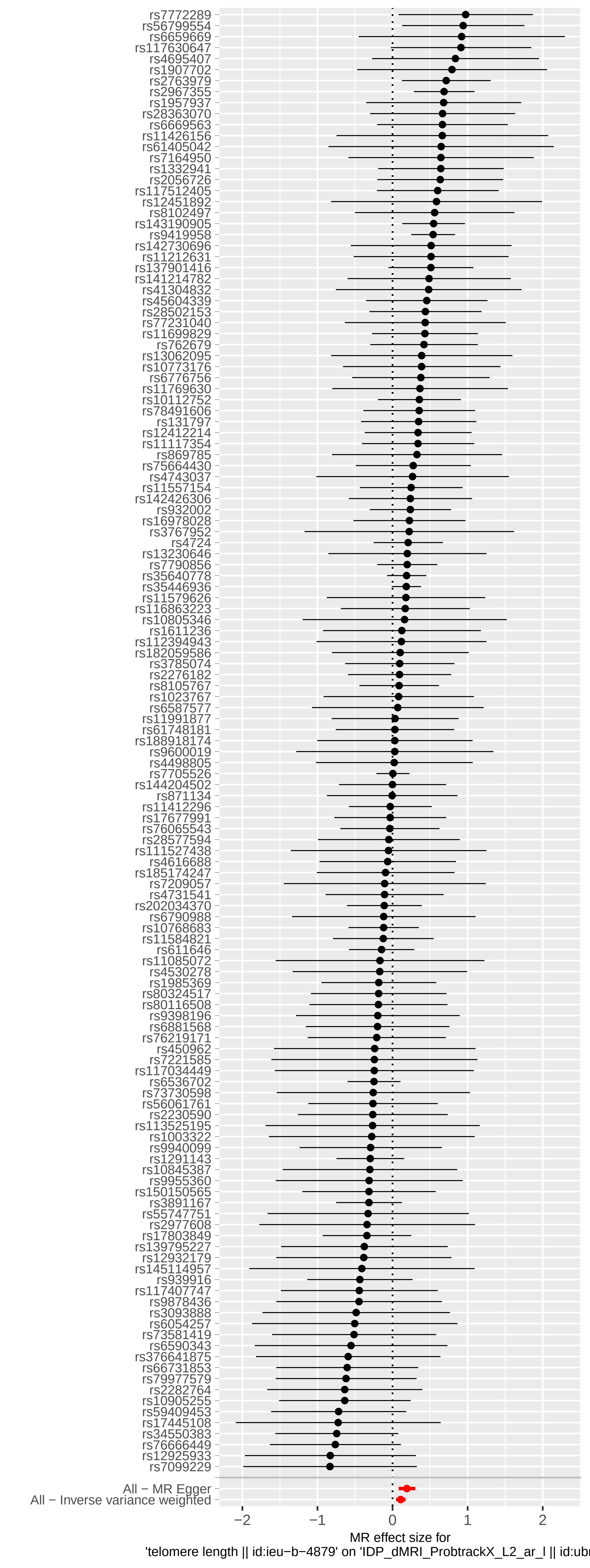

rs7772289
rs56799554
rs6659669
rs117630647
rs4695407
rs1907702
rs2763979
rs2967355
rs1957937
rs28363070
rs6669563
rs11426156
rs61405042
rs7164950
rs1332941
rs2056726
rs117512405
rs12451892
rs8102497
rs143190905
rs9419958
rs142730696
rs11212631
rs137901416
rs141214782
rs41304832
rs45604339
rs28502153
rs77231040
rs11699829
rs762679
rs13062095
rs10773176
rs6776756
rs11769630
rs10112752
rs78491606
rs131797
rs12412214
rs11117354
rs869785
rs75664430
rs4743037
rs11557154
rs142426306
rs932002
rs16978028
rs3767952
rs4724
rs13230646
rs7790856
rs35640778
rs35446936
rs11579626
rs116863223
rs10805346
rs1611236
rs112394943
rs182059586
rs3785074
rs2276182
rs8105767
rs1023767
rs6587577
rs11991877
rs61748181
rs188918174
rs9600019
rs4498805
rs7705526
rs144204502
rs871134
rs11412296
rs17677991
rs76065543
rs28577594
rs111527438
rs4616688
rs185174247
rs7209057
rs4731541
rs202034370
rs6790988
rs10768683
rs11584821
rs611646
rs11085072
rs4530278
rs1985369
rs80324517
rs80116508
rs9398196
rs6881568
rs76219171
rs450962
rs7221585
rs117034449
rs6536702
rs73730598
rs56061761
rs2230590
rs113525195
rs1003322
rs9940099
rs1291143
rs10845387
rs9955360
rs150150565
rs3891167
rs55747751
rs2977608
rs17803849
rs139795227
rs12932179
rs145114957
rs939916
rs117407747
rs9878436
rs3093888
rs6054257
rs73581419
rs6590343
rs376641875
rs66731853
rs79977579
rs2282764
rs10905255
rs59409453
rs17445108
rs34550383
rs76666449
rs12925933
rs7099229
All − MR Egger
All − Inverse variance weighted
−2
−1
0
1
2
MR effect size for
'telomere length || id:ieu−b−4879' on 'IDP_dMRI_ProbtrackX_L2_ar_l || id:ubm
